# SURE — EU Capacity for Stabilising Employment and Incomes in the Pandemic

**DOI:** 10.1007/s10272-020-0888-y

**Published:** 2020-06-07

**Authors:** László Andor

**Affiliations:** Foundation for European Progressive Studies, Rue Montoyer, 40, 1000 Brussels, Belgium

## Abstract

On 2 April 2020, the European Commission (2020) duly put forward a proposal for the creation of a European instrument for temporary Support to mitigate Unemployment Risks in an Emergency (SURE). This bold and innovative move must be welcome, but the actual profile of this new instrument requires clarification to avoid misunderstandings, false expectations and eventual disappointment.

In March 2020, European countries were suddenly overwhelmed by the COVID-19 pandemic, originating from China. The unprecedented nature of this crisis may explain why most national governments, together with leaders of the EU institutions, responded to this emergency with delays as well as inconsistency. Nevertheless, it was quickly understood that the challenge was to tame a health care, economic and social crisis simultaneously. The search for appropriate tools and strategies began.

Once it was clear that in order to slow down the epidemic, much of the economic and social activities had to stop, the risk of rapidly rising unemployment became apparent. The EU was expected not only to mobilise existing instruments, but also to quickly develop new ones. On 2 April 2020, the European Commission (2020) duly put forward a proposal for the creation of a European instrument for temporary Support to mitigate Unemployment Risks in an Emergency

## From undercurrent to paradigm shift

The SURE instrument aims to make available financial support, in the form of loans granted on favourable terms, to member states that need to mobilise significant resources to alleviate the socio-economic impact of the pandemic through short-time work (STW) schemes or similar measures. Total loans could amount to up to €100 billion. The legal basis proposed by the Commission is Article 122(1) and (2) of the Treaty on the Functioning of the European Union (D'Alfonso, 2020).

If a member state experiences a sudden severe increase in actual and planned public expenditure for the preservation of employment because of its response to the COVID-19 pandemic, it can request financial assistance under the SURE instrument to cover part of this additional expenditure. Relevant expenditure concerns the extension or creation of STW schemes or similar measures designed to protect workers from the risk of unemployment and loss of income.

Within the short period of its genesis, SURE was introduced in the context of unemployment reinsurance, but there is a very important distinction to be made. Following her nomination as EU Commission President, Ursula von der Leyen announced that during her mandate she would introduce an unemployment reinsurance scheme. This promise was included in the mission letter of two EU Commissioners: Paolo Gentiloni (Economy) and Nicolas Schmit (Jobs and Social Rights). However, SURE is not an unemployment insurance or reinsurance.

Probably the best characterisation of SURE is that it is a job insurance scheme (Fernandes and Vandenbroucke, 2020). It is a safety net for jobs, but not for the unemployed. The distinction is meaningful. In any existing unemployment insurance scheme, cash (and not a loan) is received by the unemployed individual. This will not be the case with SURE. Nevertheless, to the extent that SURE helps to lower the number of actual unemployed, the national unemployment benefit schemes will cope better.

### *Kurzarbeit* on the EU agenda

This is not the first time that the European Commission has highlighted the potential of STW solutions. It was already the case in April 2012, when the Commission put forward the Employment Package to counter the consequences of the great financial and economic crisis. By popularising STW (or in German *Kurzarbeit*) at the time of the euro area crisis, the EU did what it had been doing ever since the launch of the Lisbon Strategy: identifying best practices in the member states and sharing them through various EU processes.

On the other hand, by this clarification the EU was also moving towards defining a European labour model and creating consensus around a *hierarchy of adjustment possibilities* in the labour market for periods of economic downturns. If demand drops, adjusting through working time reduction appeared clearly superior to other options: reduction of wages, reduction of employment or reduction of the labour force by lowering the retirement age. Though very far from being uniform, Europe indeed showed some great examples of negotiated STW schemes coupled with training, for instance in Germany and Austria. This internal flexibility provides a strong basis for an economic rebound once demand picks up, creating a competitive advantage especially in comparison with the United States.

Very importantly, the 2012 Employment Package promoted internal flexibility at the time of a recession (as an alternative to external flexibility). This was seen as the royal road from an economic as well as social point of view, while not seen as a universal solution or wonder weapon. Make no mistake: the STW arrangement is a much better option than unemployment, but this option does not exist everywhere. Kurzarbeit has three main preconditions to work: first, a demand side shock after which the same economic structure can bounce back; second, strong partnership[1] between employers and trade unions; and third, financial capacity to provide support either from an unemployment fund or elsewhere. Consequently, an EU scheme focusing on Kurzarbeit would be biased for the workers who were better off in countries with stronger industrial relations, and it would leave the more precarious workers in the precarious benefit schemes of precarious countries.

Limiting EU solidarity to those workers whose jobs can be saved would raise questions. The number of unemployed is bound to rise simply because some companies will die. Furthermore, many people today have temporary contracts and in crises most of these are simply not renewed; these people will be added to the number of unemployed without being dismissed either de facto or *de jure*. Most of them would be unlikely to be considered under STW schemes, similar to the self-employed. It is therefore particularly important that the SURE initiative is open to programmes designed for the self-employed as well, but even this way it remains an exclusive support tool.

### The Multiannual Financial Framework perspective: Enhancing the social compartment

SURE does not only bring a new budgetary tool to the EU but also a new way of raising and providing resources. It does not require any upfront cash contributions from member states. To back the lending scheme, member states would commit irrevocable and callable guarantees worth €25 billion to the EU budget, with each guarantee calculated on the basis of their respective share of EU gross national income. Such a system should ensure a high credit rating, enabling the European Commission to contract borrowings on the financial markets at favourable conditions, with the purpose of on-lending them to the member state requesting financial assistance.

Until now, members states have had the possibility of financing STW schemes from the European Social Fund (ESF), but with SURE the available volumes will be enhanced by the newly created borrowing framework. SURE will help stabilise employment (and the related work income) for those whose jobs can be saved in a recession. On the other hand, the Fund for European Aid for the Most Deprived (FEAD) provides EU support for those who lack the means for buying daily food for themselves. The missing element, however, remains the EU capacity to top up national unemployment

1 On the example of Austria, Schnetzer et al. (2020) stress the importance of social partners in negotiation, and the resulting effectiveness of short-time work arrangements in the COVID-19 crisis.

insurance funds in the circumstances of a recession. While the rise of joblessness can be massive, related funding will continue to rely entirely on national resources (Andor, 2020).

Another break on the SURE model is the zero percent grant component. While borrowing under this scheme, the member state affected will have to cover administrative costs due to the need to organise the program. So the actual material help from the EU is about delayed taxation. This may still make sense, but employers will only play ball if the benefits of organising STW schemes (i.e. keeping the entire workforce on board without changing contracts) would exceed the administrative and organisational costs which would need to be shared within the country anyhow.

## The EMU perspective: Towards macroeconomic stabilisation

The size of SURE can be compared to that of the ESF, which became an ESF+ in the new Multiannual Financial Framework (MFF) proposal, with over €100 billion for seven years (Andor, 2018). This, however, is a false comparison, because the ESF provides grants, while SURE will provide loans. In order to appreciate how bold this initiative is, another comparison should apply, namely to the two instruments Jean-Claude Juncker proposed for cyclical stabilisation: the European Investment Stabilisation Function[2] and a Reform Support Programme,[3] which between them would have been able to disseminate €55 billion. Thus von der Leyen, with just a single instrument, goes well beyond Juncker, which inadvertently highlights how unserious the previous exercise was.

Volume is key for any stabilisation, and so is speed. The promotion of SURE highlights this aspect, although in this case funds can be disbursed following a procedure that involves the Council, and only if a certain conditionality[4] is met (short-time work arrangement or similar scheme). Conditionality can be a good thing; it has been rightly introduced for the ESF itself, which originally and fundamentally is not a cyclical but a structural fund. For cyclical stabilisation, conditionality at the time of delivery causes delay and by definition makes the instrument weaker.

With a clear conditionality that is strongly linked to cyclicality, SURE delivers something that has been missing from the EU architecture: a counter-cyclical fiscal capacity. In other words, this can be seen as an initial step in the direction that could eventually turn the MFF from its head to its feet, and lead to a proper stabilisation role at the community level. On the other hand, the unemployment reinsurance models that have been discussed in the past few years would also have required a coordinated improvement (in coverage, generosity, attached training services, etc.) in existing unemployment benefit schemes, which will not be the case here. Some will speak about a missed opportunity as a result.

## Getting expectations right

By creating a new instrument called SURE, the European Commission put forward a bold emergency initiative. Its size and innovative nature signal not only that the economic and the social crisis response have to go hand in hand, but also that the coronavirus crisis is calling for deeper EU integration and inviting new ideas.

While aware of its limitations, the added value of SURE has to be highlighted as European citizens are looking for help at the time of a devastating pandemic. However, it is also important to avoid overselling this tool. We may remember, for example, that when the Youth Guarantee was introduced,[5] the Commission invested a lot in explaining what it can and what it cannot do. However, the hype and the misinformation caused some damage, which is a big risk also now.

For SURE to reach its goals, clear rules are needed, as stressed by Balleer et al. (2020). Provided those are followed, SURE can be seen as a potentially cost-effective automatic stabiliser at the European level, and it may as well be the starting point for a more ambitious European unemployment reinsurance system (Vandenbroucke et al., 2020).

In any case, the macroeconomic effect of SURE will not be robust. The European capacity to limit unemployment will surely be greater than that of the United States, where the number of unemployed increased by 20 million in one month, sending the unemployment rate from 4.4% in March to 14.6% in April 2020. However, SURE will only play a minor part in the better outcome.

2 The EISF was supposed to maintain the continuity of investment projects in times of crises. However, this was supposed to happen through loans rather than transfers, in a way to compensate for interest rates potentially hiking in a turbulent period.

3 The RSP was designed to support structural reforms within the member states in line with recommendations outlined in the context of the European Semester. Apart from offering a Reform Delivery Tool and technical assistance, it also wanted to introduce a Convergence Facility to provide dedicated support to member states seeking to adopt the euro.

4 Interestingly, Alcidi and Corti (2020) consider SURE unconditional, simply because a Memorandum of Understanding is not supposed to be involved. On the other hand, since a specific action has to be taken to access the EU funding, SURE can be classified as a conditional stabiliser tool.

5 Following the December 2012 Commission proposal and April 2013 Council decision.

Perhaps the most important message here is that while structural interventions like SURE can be important, they are not substitutes for the appropriate macroeconomic policies that have to be in place to ensure the fastest possible recovery. The overall dynamics of unemployment will remain functions of the fiscal and monetary mix, while structural interventions can have mitigating effects and a positive influence on working conditions.
